# Characteristics of Bow-Tie Antenna Structures for Semi-Insulating GaAs and InP Photoconductive Terahertz Emitters

**DOI:** 10.3390/s21093131

**Published:** 2021-04-30

**Authors:** Salman Alfihed, Ian G. Foulds, Jonathan F. Holzman

**Affiliations:** 1 School of Engineering, University of British Columbia (UBC), Kelowna, BC V1V 1V7, Canada; Salfihed@alumni.ubc.ca (S.A.); Ian.Foulds@ubc.ca (I.G.F.); 2 Materials Science Research Institute, King Abdulaziz City for Science and Technology (KACST), Riyadh 11442, Saudi Arabia

**Keywords:** photoconductive THz emitter, semi-insulating THz emitter, bow-tie antenna

## Abstract

This work presents a study of photoconductive (PC) terahertz (THz) emitters based upon varied bow-tie (BT) antenna structures on the semi-insulating (SI) forms of GaAs and InP. The BT antennas have electrodes in the form of a Sharp BT, a Broad BT, an Asymmetric BT, a Blunted BT, and a Doubled BT. The study explores the main features of PC THz emitters for spectroscopic studies and sensors application in terms of THz field amplitude and spectral bandwidth. The emitters’ performance levels are found to depend strongly upon the PC material and antenna structure. The SI-InP emitters display lower THz field amplitude and narrower bandwidth compared to the SI-GaAs emitters with the same structure (and dimensions). The characterized Doubled BT structure yields a higher THz field amplitude, while the characterized Asymmetric BT structure with flat edges yields a higher bandwidth in comparison to the sharp-edged structures. This knowledge on the PC THz emitter characteristics, in terms of material and structure, can play a key role in future implementations and applications of THz sensor technology.

## 1. Introduction

Terahertz (THz) radiation lies within a distinct region of the electromagnetic spectrum, with frequencies spanning 0.1 to 10.0 THz, placing it between microwave and infrared spectra [1]. The resultant THz applications in fields such as spectroscopy and sensing are of growing interest with a trend seen towards THz on-chip sensors.

However, such miniaturization can sacrifice performance, as seen through effects such as charge carrier screening and saturation—and so it becomes necessary to optimize (and understand) the THz emission process [2]. Ultimately, an effective means of generating THz radiation is needed to meet the demands of such systems. The two most common means are photoconductive (PC) THz emission and optical rectification [3,4], and of these PC THz emission is found to yield both strong performance and an ease of integration within sensors.

Photoconductive THz emission was introduced by Auston et al. several decades ago [5] and since then many studies have put it to use [6,7]. The THz radiation is formed by a PC THz emitter when an ultrashort laser pulse with above-bandgap photon energy is incident upon its biased PC antenna, causing the photoexcited charge carriers to accelerate under the bias field [8]. The characteristics of the emitted THz radiation rely heavily upon the PC THz emitter’s features—and so there is an ongoing struggle to understand the photoexcitation process and optimize the emission. This is often done with thought to the PC THz emitter’s material and structure. The PC material for the THz emitter will ideally have a high carrier mobility, high breakdown voltage, and suitable bandgap for the pump laser’s photon energy [8]. A variety of semiconductors have been considered for this PC material, including InAs [9], InGaAs [10], GaSb [11], GaNAsSb [12], and the semi-insulating (SI) forms of GaAs [13] and InP [14]. The antenna structure has also been found to influence the PC THz emission. Specifically, the polarization dependence from different structures has been found to yield differing electric field components, which impacts the direction of charge carrier transport and the effectiveness of PC THz emission [15]. Various antenna structures have been studied in the past―and progress has been made in optimizing these structures for PC THz emitters [16]. However, such studies have also found that the characteristics of PC THz emission from the differing structures are impacted by the underlying PC material.

In this work, PC THz emitters based upon bow-tie (BT) antennas are studied with varying structures, including Sharp BT, Broad BT, Asymmetric BT, Blunted BT, and Doubled BT, on the two most forms of PC material, SI-GaAs and SI-InP. The THz field amplitude and bandwidth are found to depend upon the structure (and dimensions) as well as the material, yielding insight on the optimal implementations. Such findings can support the realization of high-performance PC THz emitters in future THz sensor technologies.

## 2. Methods

The theoretical and experimental characteristics of PC THz emitters are considered in this section with thought to the antenna structure and the underlying PC material.

### 2.1. Theoretical Characterization of Photoconductive Terahertz Emitters

**Figure 1:**
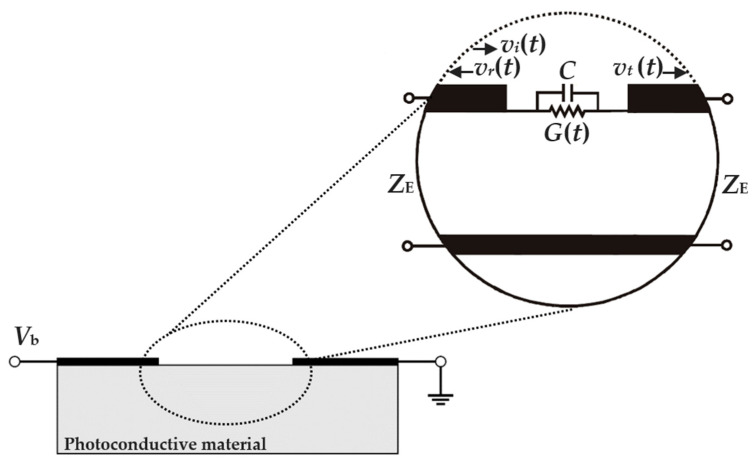
Illustration of the biased PC gap of a PC THz emitter at a bias voltage of *V*_b_. The inset shows its equivalent circuit with a gap conductance of *G(t*), gap capacitance of *C*, and electrodes with transient incident, reflected, and transmitted voltage waveforms of *v*_i_(*t*), *v*_r_(*t*), and *v*_t_(*t*), respectively.

The functioning of a PC THz emitter can be understood in terms of charge carrier transport in its PC material, due to the acceleration of the photoexcited charge carriers, and charge redistribution on its electrodes, as voltage on the electrodes varies to accommodate the evolving electric field in the PC gap. These effects can be modelled by a conduction current, according to a time-varying gap conductance of *G*(*t*), and a displacement current, as defined by a gap capacitance of *C*, respectively. Such a construct leads to the equivalent circuit of a PC gap shown in Figure 1 with a bias voltage of *V*_b_ and a parallel connection of its conductor and capacitor. For the conduction current, the photoexcitation yields a time-varying conductivity of

*σ(t)* = *n*_*s*_*(Φ_p_)q*_*e*_*μ*_*n*_*(Φ_p_)* (1)

where *n*_s_(*Φ*_p_) is the electron surface charge density at a pump fluence (i.e., energy per unit area) of *Φ*_p_, *q*_e_ = 1.602176634 × 10^−19^ C is the electronic charge, and *μ*_n_(*Φ*_p_) is the pump-fluence-dependent electron mobility. The gap conductance, *G*(*t*), can then be approximated by integrating this conductivity over the transverse width and depth of photoexcitation in the PC gap. The gap conductance, *G*(*t*), and the gap capacitance, *C*, can be linked to the resistance-capacitance (RC) time constant of

*τ*_RC_ = 2*Z*_E_*C*/(2*Z*_E_*CG*(*t*) + 1) (2)

where *Z*_E_ is the antenna/transmission line impedance. It can be seen here that the conductivity, conductance, and conduction current all increase in proportion to the mobility. For the displacement current, the evolving electric field in the PC gap causes a redistribution of charge on the electrodes, yielding the transient incident, *v*_i_(t), reflected, *v*_r_(t), and transmitted, *v*_t_(t), voltage waveforms shown in the inset of Figure 1. This redistribution will ideally occur on a subpicosecond timescale, to allow the antenna to radiate into the THz spectrum, but this can only manifest if the structure has a sufficiently small gap capacitance, *C*. The τ_RC_ defines the shape and duration of the THz pulse, with shorter τ_RC_ values producing shorter pulse durations (in the time-domain) and wider bandwidths (in the frequency-domain). Ultimately, it can be concluded that both the structure and material of the PC gap play a role in defining the THz field amplitude and spectral bandwidth. The bandwidth is defined in the frequency-domain with respect to the noise floor. It is the difference between the highest measurable frequency (when the signal drops down into the noise floor) and the lowest measurable frequency (where the signal rises up out of the noise floor).

### 2.2. Experimental Characterization of Photoconductive Terahertz Emitters

**Figure 2:**
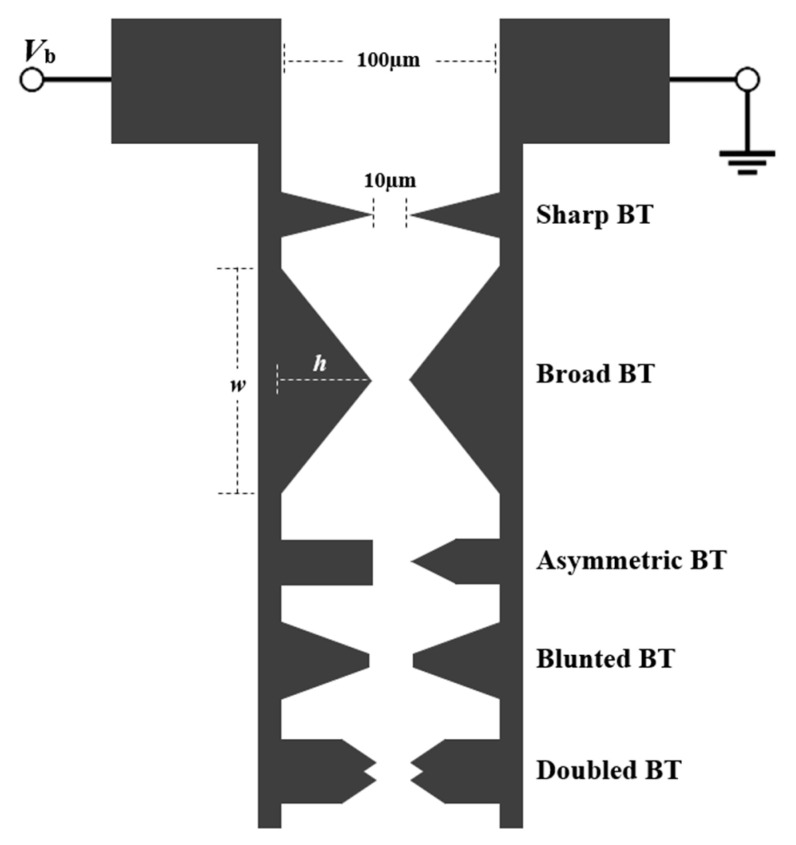
The five investigated structures, including Sharp BT, Broad BT, Asymmetric BT, Blunted BT, and Doubled BT antenna structures.

The PC THz emitters analysed in this work were fabricated via sputtering as Cr/Au metallization at a thickness of 25/75 nm. Conventional photolithography was then used to pattern the metal via bilayer lift-off with a mask aligner (OAI Model 204). The structures were implemented with a fixed 10-µm PC gap and a fixed 100-µm outer spacing between the two electrodes in the form of Sharp BT, Broad BT, Asymmetric BT, Blunted BT, and Doubled BT antenna structures, as shown in Figure 2. The fabricated BT antennas were characterized using an SEM system (Tescan Mira 3 XMU Scanning Electron Microscope) with Backscattered-Electron (BSE) imaging, at 1000× magnification and 20.0 kV.

**Figure 3:**
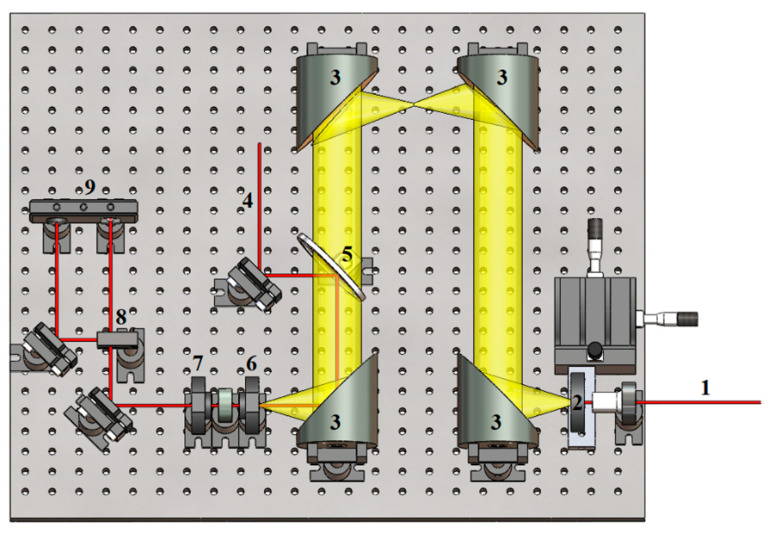
Illustration of THz-TDS used to characterize the PC THz emitter, pump beam (1) is focused onto the PC THz emitter (2), the generated THz beam (yellow) is focused and steered via parapolice mirror-(3). The probe beam (4, red) is overlapped with the THz beam via pellicle beam-splitter (5), modulated via electro-optic crystal (6) and quarter waveplate (7), separated by a polarizing beam-splitter (8) into two orthogonal polarizations beams, and the power difference between these two beams is measured by a differential photodetector (9).

Characterizations of the PC antennas were carried out using a THz-time-domain spectroscopy (THz-TDS) system shown in Figure 3. An ultrafast pulsed laser (Spectra Physics-Mai Tai HP) was used with an 800-nm wavelength, a 70-fs pulse duration, and an 80-MHz reputation rate. The ultrashort laser pulses delivered by the system were split into separate pump and probe beams. The pump beam (with an average power of *P*_P_ = 0.6 W) was focused on the biased PC gap (with a bias voltage of *V*_b_ = 7.5 V), such that the focal spot roughly covered the 10-µm gap. In this way, the pump-induced charge carriers would accelerate in the bias field to produce the desired THz radiation. The THz radiation was then sampled by the probe beam in an electro-optic (EO) crystal, in the form of a 0.5-mm-thick ZnTe <110> crystal. The THz-induced polarization rotation on the probe beam was then extracted via polarization-sensitive optics [4]. The THz field amplitude, *E*_THz_(*t*), was recorded as a function of the pump-probe delay time, *t*, with the result Fourier transformed to obtain the THz spectral amplitude, *E*_THz_(*f*), as a function of frequency, *f*. In general, the spectrum can be subject to absorption and dispersion via phonon modes in the EO crystal [17]. However, for this study on relative differences in THz amplitudes and bandwidths, for the various structures and materials, the absorption and dispersion are common to all of the measurements and are therefore of little consequence.

## 3. Results and Discussion

**Figure 4:**
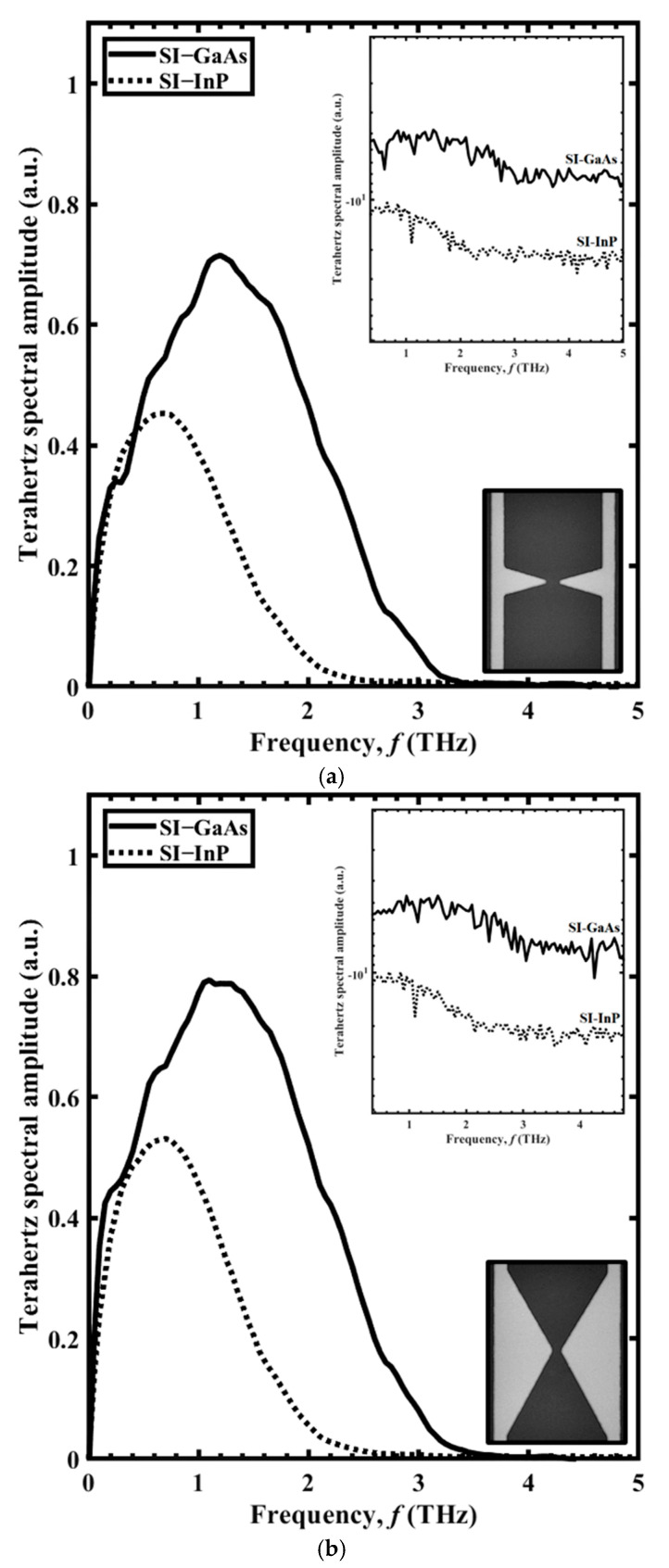
The THz spectral amplitude as a function of frequency, *f,* for PC THz emitters incorporating SI-GaAs (solid lines) and SI-InP (dotted lines) in the form of the (**a**) Sharp BT and (**b**) Broad BT. The THz spectral amplitudes are shown on a log scale in the upper insets, and the two structures are shown as SEM images in the lower insets, with lighter colour denoting metal.

Results for the THz spectral amplitude as a function of frequency, *f*, are shown in Figure 4 for the BT antennas with (a) Sharp BT and (b) Broad BT antenna structures. The spectra are given for SI-GaAs (black line) and SI-InP (dotted line) as the PC material, with the THz spectral amplitudes shown on a log scale in the upper insets and the scanning electron microscope (SEM) images of the structures shown in the lower insets. Each structure can be envisioned as two isosceles triangles, with their tips meeting at the PC gap, where each triangle has a base width of *w* (the longest dimension of the triangle) and an overall height of *h* (from the tip to the electrode). Thus, the Sharp BT and Broad BT structures have height-to-width ratios of *h*/*w* ≈ 1.8 and 0.28, respectively, at roughly a factor of six difference. For the Sharp BT structure, with SI-GaAs and SI-InP, the (maximum normalized) THz field amplitudes are 0.66 and 0.49 respectively, and the bandwidths are 3.4 and 2.5 THz, respectively. For the Broad BT structure, with SI-GaAs and SI-InP, the (maximum normalized) THz field amplitudes are 0.83 and 0.62, respectively, and the bandwidths are 3.5 and 2.6 THz, respectively. The results show that the Broad BT has a larger amplitude and bandwidth compared to the Sharp BT. The improvement of the radiated THz field with respect to BT antenna dimensions was reported by Yang et al. [18] when the authors found a correlation between BT tip-length and the radiated THz field strength and bandwidth. We attribute this to the greater photocurrent in the transverse direction of the Broad BT, which results in a larger amplitude. The bandwidths resulted from these two structures are related to the broader THz pulse width as described by Tani et al. [16]. The improvement of the bandwidth is associated with the smaller capacitance of the Broad BT over the Sharp BT, which results in a shorter *τ*_RC_ according to (2) and producing shorter THz pulse durations and ultimately wider bandwidth.

**Figure 5:**
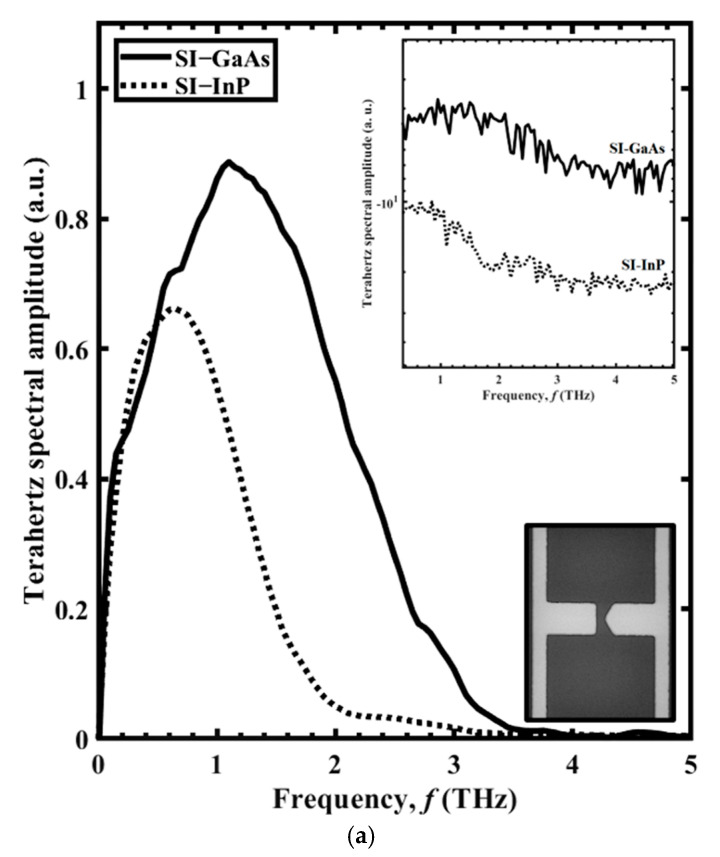

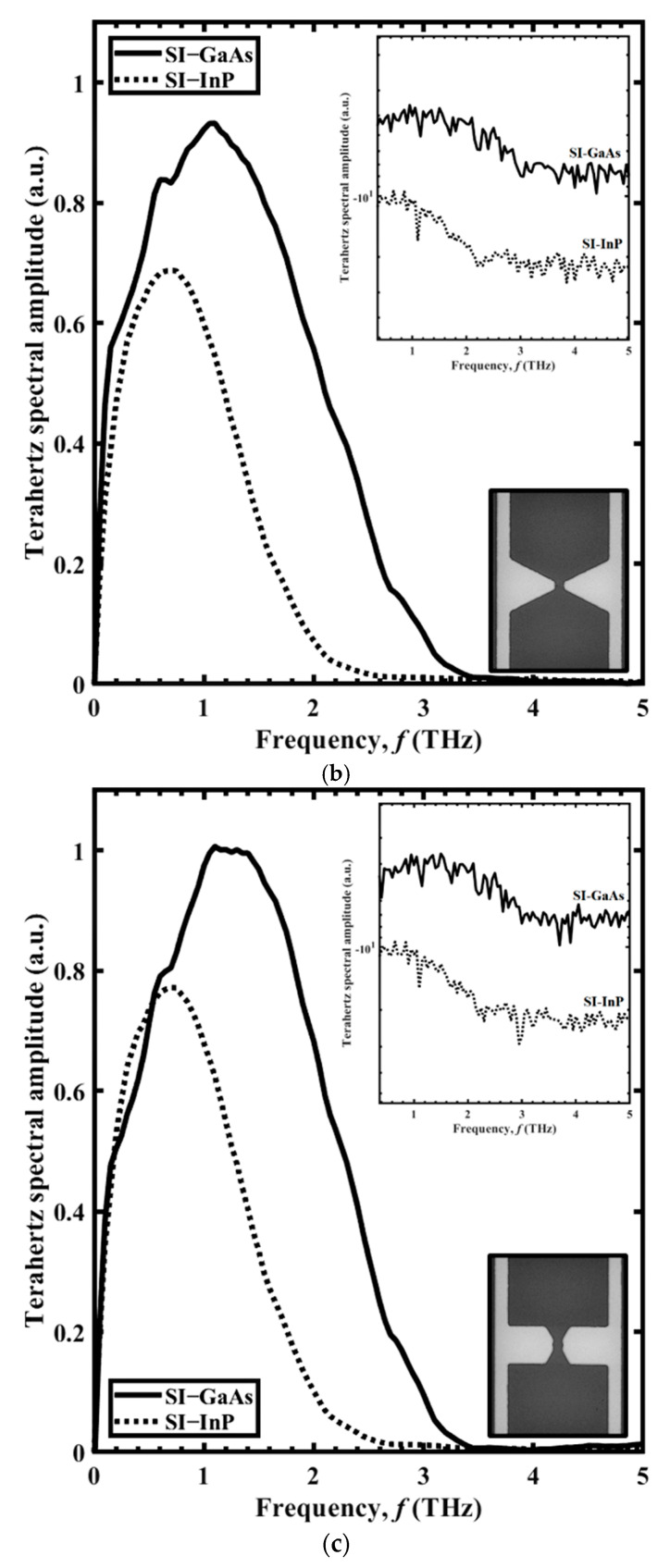
The THz spectral amplitude as a function of frequency, *f,* for PC THz emitters incorporating SI-GaAs (solid lines) and SI-InP (dotted lines) in the form of (**a**) Asymmetric BT, (**b**) Blunted BT, and (**c**) Doubled BT structures. The THz spectral amplitudes are shown on a log scale in the upper insets, and the three structures are shown as scanning electron microscope (SEM) images in the lower insets, with lighter colour denoting metal.

Results for the THz spectral amplitude as a function of frequency, *f*, are shown in Figure 5 for the bow-tie antennas with (a) Asymmetric BT, (b) Blunted BT, and (c) Doubled BT structures. The spectra are given for SI-GaAs (black line) and SI-InP (dotted line) as the PC material, with the THz spectral amplitudes are shown on a log scale in the upper insets and the scanning electron microscope (SEM) images of the structures shown in the lower insets. For the Asymmetric BT structure, with SI-GaAs and SI-InP, the normalized THz field amplitudes are 0.85 and 0.63, respectively, and the bandwidths are 3.7 and 3.2 THz, respectively. For the Blunted BT structure, with SI-GaAs and SI-InP, the normalized THz field amplitudes are 0.89 and 0.72, respectively, and the bandwidths are similar to those of the Asymmetric BT structure at 3.5 and 2.8 THz, respectively. To accentuate the effects of electric field singularities, the Doubled BT structure is considered. It has four sharp edges coming together within the PC gap. For the Doubled BT structure, with SI-GaAs and SI-InP, the (maximum normalized) THz field amplitudes are 1.0 and 0.75, respectively, and the bandwidths are 3.4 and 2.8 THz, respectively. The normalized THz field amplitude then increases from 0.85 for the Asymmetric BT structure to 1.0 for the Doubled BT structure. It is proposed here that the increase in bias field, due to its localization, raises the THz field amplitude, in agreement with Cai et al. [15]. However, the spectral bandwidth for the Asymmetric BT is greater than those of the Blunted BT and Doubled BT structures, which we attribute to its lower capacitance.

It worth noting that these results were obtained with a pump laser spot diameter of roughly 10 µm, which matches the size of the PC gap. Spot sizes that are far larger than the PC gap yield fewer charge carriers in the gap, reducing the photogenerated current and PC THz emission. In contrast, spot sizes that are far smaller than the PC gap do not allow for full conduction across the gap, preventing the photocurrent from coupling to the antenna and reducing the PC THz emission.

**Figure 6:**
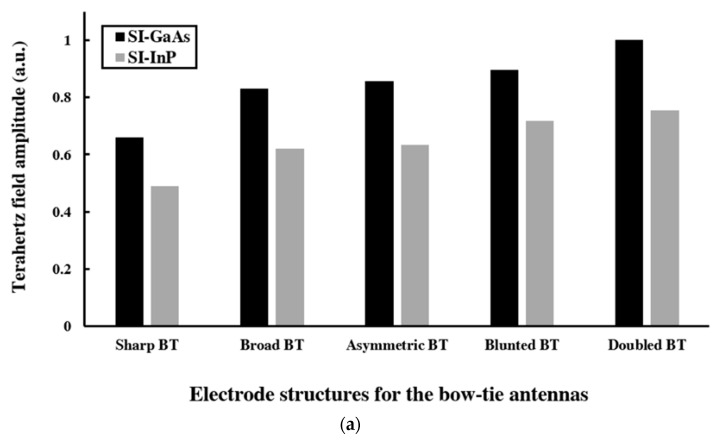

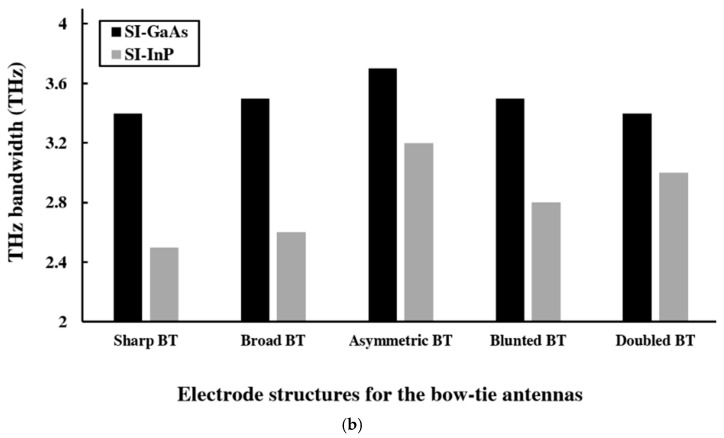
The (**a**) THz field amplitude and (**b**) THz bandwidth for PC THz emitters based upon SI-GaAs (black) and SI-InP (grey) with BT antennas having the Sharp BT, Broad BT, Asymmetric BT, Blunted BT, and Doubled BT structures.

The overall results for the THz field amplitude of the all PC THz emitters are shown in Figure 6a for the varying forms of BT antennas with SI-GaAs (black) and SI-InP (grey) materials. Clearly, the THz field amplitudes for SI-GaAs are higher than those of SI-InP for each of the structures. We attribute this improved performance for SI-GaAs to its higher electron mobility and thus its greater conduction current. (The THz field amplitude, *E*_THz_, is proportional to the integral of the conduction current density across the PC gap, as described in our prior work [19,20].) We also note that the bandgap energies of SI-GaAs and SI-InP are 1.43 eV and 1.34 eV, respectively, such that the pump photons, with energies of 1.55 eV, deposit charge carriers closer to the conduction band minimum of SI-GaAs, as compared to SI-InP. This decreases the excess energy of the hot electrons in SI-GaAs and lessens their pump-induced reduction in electron mobility [20]. For SI-InP, the hot electrons are deposited higher in the conduction band, which demands time (typically on a picosecond duration) for energy relaxation to the conduction band minimum where they attain their maximal mobility.

The overall results for the THz bandwidth of the all PC THz emitters are shown in Figure 6b. Notably, all the obtained results exhibit a wider bandwidth of SI-GaAs over SI-InP. We attribute this difference (again) to the greater mobility of SI-GaAs. The greater mobility increases the conductivity in (1), and thus the conductance, *G*(*t*), which reduces the *τ*_RC_ in (2).

The presented results of the PC THz emitters were obtained using a pulsed laser system with an 800-nm pump wavelength, a 70-fs pulse duration, and an 80-MHz reputation rate. The 800-nm pump laser wavelength was chosen here due to our studies of PC THz emitter performance versus pump laser wavelength spanning 790–870 nm. The THz field amplitude was found to be relatively high and flat for wavelengths below 830 nm and 850 nm in SI-GaAs and SI-InP, respectively, with a sharp drop above these wavelengths (due to the photon energies dropping below the conduction band edge). In this work, the 800-nm laser wavelength was chosen to be sufficiently far from this drop, while having a common wavelength for all of the characterizations. With respect to the laser pulse duration, the presented results were found to be limited by charge carrier transport and capacitance in the gaps. Thus, we do not expect significant changes in the results for shorter pulse durations. In contrast, longer pulse durations could ultimately slow the charge carrier photoexcitation and reduce the bandwidth. With respect to the repetition rate, we would not expect significant changes in the results for lower repetition rates. This is because lower repetition rates have periods between laser pulses that are longer than the charge carrier lifetimes of SI-GaAs and SI-InP. In contrast, higher repetition rates could leave pre-populated/steady-state charge carriers within the PC THz emitter, which would increase its ohmic loss and lessen its amplitude.

## 4. Conclusions

In this work, a variety of BT antenna structures with different characteristics, based upon SI-GaAs and SI-InP were investigated as PC THz emitters. It was found that the SI-GaAs emitters provide higher THz field amplitude over SI-InP due to their higher carrier mobility. The Doubled BT structure, with its pronounced electric field singularities, yielded the highest THz field amplitude. However, this came at the expense of a greater capacitance and lower bandwidth. The greatest THz bandwidth resulted from the Asymmetric BT structure as a result of its lower capacitance. Such findings can support the understanding and optimization of PC THz emitters in future realizations of THz sensor technologies.
